# Resorcinol and Related Simple Phenolic Compounds Increase Astrocyte Survival in a Glutamate-Induced Excitotoxicity Model

**DOI:** 10.3390/jox16040142

**Published:** 2026-07-31

**Authors:** José M. Nájera-Maldonado, Paola Molina-Manzano, Eugenia Flores-Alfaro, Mónica Espinoza-Rojo, Isela Parra-Rojas, Patricia Alvarez-Fitz, Mónica Lamas, Ricardo Salazar, Mónica Ramírez

**Affiliations:** 1 Facultad de Ciencias Químico-Biológicas, Universidad Autónoma de Guerrero, Chilpancingo 39087, Mexico; josenajera@uagro.mx (J.M.N.-M.); 10103351@uagro.mx (P.M.-M.); eugeniaflores@uagro.mx (E.F.-A.); monicaespinoza@uagro.mx (M.E.-R.); iselaparra@uagro.mx (I.P.-R.); 2 Secretaría de Ciencia, Humanidades, Tecnología e Innovación (SECIHTI), Universidad Autónoma de Guerrero, Chilpancingo 39087, Mexico; palvarezfi@secihti.mx; 3 Departamento de Farmacobiología y Centro de Investigación Sobre el Envejecimiento, Centro de Investigación y de Estudios Avanzados (CINVESTAV), Unidad Sede Sur, Mexico City 14330, Mexico; mlamas@cinvestav.mx

**Keywords:** glutamate (Glu), excitotoxicity, astrocytes, cell death, glioprotection, simple phenolic compounds, ortho-, meta-, para-configurations, resorcinol

## Abstract

Simple phenolic compounds can act as antioxidants or prooxidants depending on their structure and environmental conditions. Glutamate-induced excitotoxicity leads to neurodegeneration, astrocyte dysfunction, oxidative stress, and neuroinflammation. This study examined six phenolics: catechol, phloroglucinol, resorcinol, pyrogallol, hydroquinone, and hydroxyquinol, assessing their effects on astrocyte survival in a glutamate-excitotoxic model. Mouse astrocytes (C8-D1A) were exposed to 20 mM glutamate, with phenolics added before, during, or after treatment. Cell viability, morphology, nuclear condensation, ROS levels, and inflammatory cytokines were measured using the MTT assay, microscopy, CellROX, and RT-qPCR. Simultaneously, treatment improved astrocyte survival and preserved morphology, whereas pre- or post-treatment did not confer protection and worsened toxicity at higher doses. Resorcinol showed the strongest protective effect, followed by hydroxyquinol and hydroquinone at lower doses. Resorcinol and hydroxyquinol decreased ROS levels, whereas pyrogallol and hydroquinone increased cell survival without reducing ROS. All four lowered *IL-6* and *TNF-α*. These findings demonstrate that the effects of phenolics, whether protective or toxic, depend on concentration, structure, and timing, underscoring their dual role in excitotoxic environments. They also identify resorcinol as a promising candidate for further preclinical study.

## 1. Introduction

Glutamate (Glu) is a major intracellular amino acid in the brain, serving as a key neurotransmitter in CNS communication and synaptic plasticity. It is a precursor to metabolites and other neurotransmitters, including GABA and N-acetyl-aspartyl-glutamate. Together, glutamatergic and GABAergic signaling maintains the balance between excitatory and inhibitory neuronal communication [1]. Excessive Glu leads to excitotoxicity, caused by overactivation of N-methyl-D-aspartate (NMDA) receptors. This results in oxidative stress, calcium imbalance, impaired protein degradation, ER stress, DNA damage, mitochondrial dysfunction, and increased neuronal death [2].

As we age, some brain regions become more vulnerable to cell death, increasing the risk of neurodegenerative diseases. For example, neuronal loss in the hippocampus is linked to memory decline. Additionally, Glu-mediated excitotoxicity is a hallmark of several neurodegenerative conditions, including stroke, Alzheimer’s, and Parkinson’s diseases [3].

Astrocytes are the most abundant glial cells in the CNS; they not only provide structural and metabolic support to neurons but also play a dual protective or cytotoxic role by secreting neurotrophic factors or releasing proinflammatory cytokines. They are also central to maintaining brain redox homeostasis and protecting neurons from oxidative damage. However, under pathological conditions such as excitotoxicity, excessive ROS production can overwhelm astrocytic antioxidant defenses, leading to oxidative damage to biomolecules. Oxidative DNA lesions impair astrocyte function, disrupt homeostasis, and contribute to the progression of neurodegenerative diseases. Therefore, preserving astrocyte integrity and limiting oxidative damage are important therapeutic targets in neuroprotection [4,5,6].

Phenolic compounds are plant secondary metabolites in foods and medicinal plants. They have hydroxyl groups on aromatic rings, enabling redox reactions. Their antioxidant activity involves donating hydrogen or electrons to reactive species, forming phenoxyl radicals. These compounds also prevent oxidative damage by chelating metals, inhibiting lipid peroxidation, and modulating antioxidant defenses [7,8]. However, their protective effects in vivo cannot be attributed solely to direct radical scavenging because dietary polyphenols undergo extensive intestinal and hepatic metabolism and generally reach low concentrations in plasma and tissues. Their antioxidant effects also involve modulating redox-sensitive signaling pathways and inducing endogenous defenses, including enzymes regulated by the Nrf2/ARE pathway [9].

Despite their antioxidant properties, phenolic compounds can act as pro-oxidants under certain conditions. After donating an electron or a hydrogen atom, phenoxyl radicals can undergo further oxidation to form semiquinone and quinone intermediates, which may participate in redox cycling, generating O_2_^−^ and H_2_O_2_. Polyphenols can also reduce Fe^3+^ or Cu^2+^, generating redox-active Fe^2+^ or Cu^+^ that promote Fenton or Fenton-like reactions when H_2_O_2_ is available. The balance between antioxidant and pro-oxidant activity depends on chemical structure, concentration, pH, the availability of transition metals, and the surrounding redox environment. Phenolic compounds containing catechol or pyrogallol moieties are particularly susceptible to oxidation and may act as antioxidants at low or moderate concentrations but become pro-oxidants at higher concentrations or under metal-rich, oxidizing conditions. This dual redox activity influences their biological effects: controlled ROS generation may activate adaptive antioxidant responses, whereas excessive ROS production can cause oxidative damage [7,10].

Because of their antioxidant, anti-inflammatory, and cytoprotective properties, phenolic compounds have attracted considerable interest as potential neuroprotective agents. Growing evidence indicates that many phenolic compounds can attenuate glutamate-induced excitotoxicity by mitigating oxidative stress, neuroinflammation, mitochondrial dysfunction, and apoptotic signaling [11,12,13].

Simple phenols (one ring with two or three substitutions) exhibit properties that make them suitable as brain-based therapeutic agents; they are low-molecular-weight phenols that permeate the intestinal tract and the blood–brain barrier (BBB) [14,15,16]. For example, pyrogallol has been reported to inhibit the aggregation of alpha-synuclein, a protein implicated in Parkinson’s disease, Lewy body dementia (LBD), and multiple system atrophy (MSA) [17].

Several studies show that simple phenols can regulate multiple signaling pathways, some of which may be linked to brain processes. Pyrogallol can inhibit the phosphatidylinositol 3-kinase (PI3K)/protein kinase B (AKT) pathway in an miR-134-dependent manner [17]. A hydroquinone derivative (di-terpene para-hydroquinone) activates the Nrf2/ARE system, inducing expression of *HO-1* and *NQO1* [18]. Hydroxyquinol attenuates iNOS mRNA expression and induces activation of p38 MAPK and NF-κB in LPS-stimulated murine BV-2 microglia [19]. Other simple phenols, such as resorcinol [20], pyrogallol [21], and hydroquinone [22], have demonstrated antioxidant and anti-inflammatory effects.

Growing evidence highlights the neuroprotective role of phenolic compounds, primarily polyphenols such as flavonoids, stilbenes, and phenolic acids [11,13,23]. While StructureActivity Relationship (SAR) studies have examined how hydroxyl patterns influence antioxidant activity, their role in glioprotection during Glu excitotoxicity is less well understood. Past research often tested individual phenolics in separate models, complicating comparisons of their effects.

Therefore, this study investigates how the position and number of hydroxyl groups in simple phenolic compounds affect antioxidant, prooxidant, anti-inflammatory, and protective properties in astrocytes. Its goal is to address existing knowledge gaps and enhance understanding of SAR. The study examines the glioprotective effects of catechol, phloroglucinol, resorcinol, pyrogallol, hydroquinone, and hydroxyquinol in an in vitro model of Glu excitotoxicity using C8-D1A astrocytes. These benzene derivatives differ in the arrangement of hydroxyl groups, and we hypothesized that the position and number of hydroxyl groups determine their protective effects in astrocytes by differentially modulating cell viability, oxidative stress, and the neuroinflammatory response.

## 2. Materials and Methods

### 2.1. Compounds, Reagents, and Antibodies

**Figure 1:**
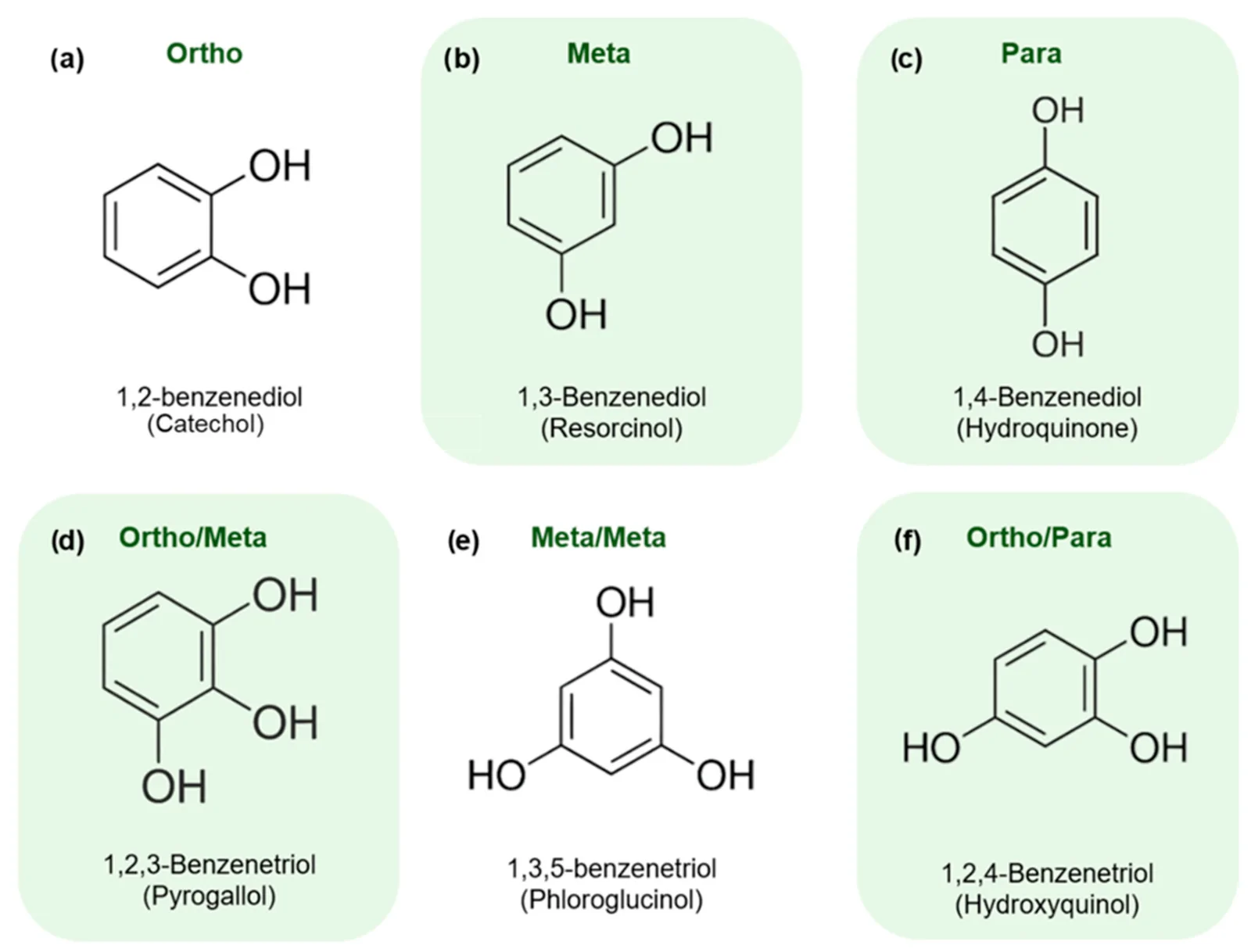
Simple phenol hydroxyl configurations. Simple phenolic structures with (**a**–**c**) two or (**d**–**f**) three hydroxyl groups, displaying (**a**,**d**,**f**) ortho-, (**b**,**d**,**e**) meta-, and (**c**,**f**) para-substitution patterns. (**a**) catechol, (**b**) resorcinol, (**c**) hydroquinone, (**d**) pyrogallol, (**e**) phloroglucinol, and (**f**) hydroxyquinol. Green-highlighted structures were selected for further evaluation.

The reagents used in cell culture were obtained from Gibco™, Thermo Fisher Scientific (Waltham, MA, USA): Dulbecco’s Modified Eagle Medium (DMEM)/F12 (#1250062), antibiotic–antimycotic (#15240062), and 0.25% trypsin-EDTA (#25200-072). Fetal bovine serum (FBS) was obtained from 90020-Byproductos (Guadalajara, Mexico). Dimethyl sulfoxide (DMSO) was obtained from Meyer (Mexico City, Mexico). The six phenolic compounds used were obtained from Sigma-Aldrich (St. Louis, MO, USA): resorcinol (#398012), pyrogallol (#P0381), hydroquinone (#H9003), hydroxyquinol (#B4001), catechol (#C9510), and phloroglucinol (#P3502). These molecules represent simple phenolic structures with two or three hydroxyl groups displaying ortho-, meta-, and para-substitution patterns (Figure 1). 3-[4,5-dimethylthiazol-2-yl]-2,5 diphenyl tetrazolium bromide (MTT; #M2128), 2,2-diphenyl-1-picrylhydrazyl (DPPH; #D9132), 1-methyl-2-phenylindole (MPI; #404888), 1,1,3,3-tetramethoxypropane (#108383), 2,4-dinitrophenylhydrazine (DNPH; #D199303), 2′,7′-dichlorodihydrofluorescein diacetate (DCFH-DA) probe (D6883), ββ-NADPH (NADPH; #N1630), fluoroshield with DAPI (#F6057), and glutamate (#G1251) were also obtained from Sigma-Aldrich. Reagents for protein quantification and gene expression, including the Bradford reagent (#B6916), iScript™ Advanced cDNA Synthesis Kit (#1725037), and SsoAdvanced™ Universal SYBR Green Supermix (#1725271), were obtained from Bio-Rad (Hercules, CA, USA), and TRIzol reagent (#15596026) was obtained from Invitrogen. Reagents and antibodies for Western blot were Blotting-Grade Blocker (1706404) from Bio-Rad; primary p-Akt (ab192623) and total Akt (ab179463) from Abcam (Cambridge, UK); p-Erk 1/2 (9101S) from Cell Signaling (Danvers, MA, USA); and β-actin (sc-81178) from Santa Cruz Biotechnology (Dallas, TX, USA). HRP-coupled anti-mouse (115-035-003) and anti-rabbit (111-035-003) secondary antibodies were obtained from Jackson ImmunoResearch (West Grove, PA, USA). Pierce^®^ ECL Western Blotting Substrate (RD231095) and CellROX™ Green Reagent (C10444) were obtained from Thermo Fisher Scientific (Waltham, MA, USA).

### 2.2. Astrocyte Cell Line Culture, Glutamate-Induced Excitotoxicity Model, and Phenolic Treatments

C8-D1A murine astrocytes were selected because they provide a homogeneous, well-characterized immortalized astrocytic model for investigating both physiological astrocyte functions and astrocyte involvement in neurodegenerative processes. Their homogeneous phenotype, reproducibility, and compatibility with small-molecule screening make them suitable for comparative pharmacological studies of glutamate-induced oxidative and inflammatory responses [24]. The C8-D1A cell line (CRL-2541™), obtained from ATCC (Manassas, VA, USA), was maintained in DMEM-F12 (1:1) supplemented with 5% FBS, 1% streptomycin, and an antifungal agent at 37 °C in a humidified incubator with 5% CO_2_. For the excitotoxicity assay, cells were exposed to Glu concentrations ranging from 0.62 to 80 mM for 24 h; cell viability and imaging confirmed the model, as described by Xin et al. [25]. A 20 mM Glu dose was chosen to induce excitotoxicity. Although basal extracellular glutamate concentrations in the CNS are generally maintained within the nanomolar to low-micromolar range, substantially higher concentrations are typically required to induce measurable toxicity in isolated cell cultures. In the present study, 20 mM glutamate was selected based on preliminary concentration–response experiments demonstrating reproducible oxidative injury in C8-D1A astrocytes under our experimental conditions. This concentration should therefore be interpreted as an in vitro injury paradigm. Phenolic compounds were dissolved in DMEM/F12 and tested at concentrations ranging from 0.19 to 200 µg/mL. They were initially evaluated using a two-fold serial dilution across a range that included both non-cytotoxic and cytotoxic concentrations to establish concentration–response relationships. In pre-treatment, phenols were applied 24 h before Glu exposure; in co-treatment, phenols and 20 mM Glu were added simultaneously for 24 h; and in post-treatment, cells were first exposed to 20 mM Glu for 24 h, and then phenols were added for another 24 h. All treatments were performed in medium supplemented with 1% FBS.

### 2.3. Cell Viability Assay

Cell viability was evaluated with the MTT assay. In brief, 1 × 10^4^ C8-D1A cells were plated in 96-well plates. After 24 h of growth, the cells were treated with Glu and/or phenolic compounds as previously described. The cells were then incubated in the dark with 5 mg/mL MTT for three hours at 37 °C. The formed formazan crystals were dissolved in DMSO, and absorbance was measured at 545 nm using a Multiskan FC Microplate Reader (Thermo Fisher Scientific, Shanghai, China). Cell viability percentage was calculated by dividing the absorbance of treated samples by that of the untreated control and then multiplying by 100.

### 2.4. Protein Quantification and Sample Preparation

For biochemical assays and Western blot analysis, astrocytes were harvested and lysed. Cells intended for oxidative stress evaluations (ROS, GPx, MDA, and PCO) were resuspended in phosphate-buffered saline (PBS) and lysed on ice with a probe sonicator using 3 cycles of 20 s at 40% amplitude to prevent protein degradation. For Western blotting, cells were lysed with a chemical lysis buffer supplemented with protease and phosphatase inhibitors. In all cases, total protein concentration was determined by the Bradford assay, using bovine serum albumin (BSA) as the standard. Absorbance was measured at 595 nm, and protein concentrations were used to normalize all subsequent biochemical measurements.

### 2.5. Radical Scavenger Assays

The radical-scavenging activity of the four selected phenols was evaluated using the 2,2-diphenyl-1-picrylhydrazyl (DPPH) and 2,2-azino-bis(3-ethylbenzothiazoline-6-sulfonic) acid (ABTS) assays. Briefly, a 50-μL aliquot of each phenol (0.19 to 200 µg/mL) was incubated at room temperature in the dark with 150 μL of a 0.004% DPPH or ABTS working solution. For the DPPH assay, the mixture was incubated for 30 min, and absorbance was measured at 517 nm. For the ABTS assay, absorbance was measured at 734 nm after a 6-min incubation. Ultrapure water and ethanol served as the blank control. All measurements were performed using a Multiskan FC microplate reader (Thermo Fisher Scientific, Shanghai, China).

For both methods, the radical-scavenging inhibition percentage was calculated using the following equation: Inhibition (%) = (A control − A sample/A control) × 100. Where A control is the absorbance of the radical control, and A sample is the absorbance of the radical in the presence of the antioxidant. The IC50 value, defined as the concentration required to scavenge 50% of the radicals, was determined by non-linear regression analysis. Data were fitted to a sigmoidal dose–response curve using GraphPad Prism 9.4 as shown in Supplementary Table S1.

### 2.6. Evaluation of Intracellular ROS Production

Reactive oxygen species (ROS) production was evaluated using two fluorescent probes. For resorcinol and hydroxyquinol, the CellROX™ assay was used. Briefly, astrocytes were seeded in 24-well plates (1 × 10^5^ cells/well) and treated with 20 mM Glu and phenolic compounds for 3 h. After treatment, 5 µM CellROX™ was added, and cells were incubated for 30 min at 37 °C. For pyrogallol and hydroquinone, the DCFH-DA probe was used to avoid potential spectral interference or direct chemical interactions between these tri- and di-phenols and the fluorophore. Separating the probes by chemical configuration prevents autooxidative crosstalk outside the cell, ensuring accurate quantification of intracellular ROS. In this case, cells were incubated in the dark with 10 µM DCFH-DA for 30 min at 37 °C. For both evaluations, cells were detached with trypsin, transferred to microtubes, and fluorescence intensity was measured using a GloMax^®^ Jr (Promega, Madison, WI, USA) detection system. All results were expressed as Relative Fluorescence Units (RFU) and normalized to total protein concentration.

### 2.7. Glutathione Peroxidase (GPx) Activity

To determine GPx activity, astrocyte lysates obtained by sonication were incubated for 5 min in a redox medium (50 mM PBS, pH 7.0; 1 mM EDTA; 1 mM sodium azide; 0.2 mM NADPH; 1 U/mL glutathione reductase; and 1 mM reduced glutathione) at 37 °C. Absorbance was measured at 340 nm for 3 min, with readings taken at 1-min intervals using a NanoDrop spectrophotometer (Thermo Fisher Scientific, Wilmington, DE, USA). GPx activity was calculated using the NADPH extinction coefficient and normalized to total protein content.

### 2.8. Lipid Peroxidation (MDA) and Protein Carbonyl (PCO) Content

Malondialdehyde (MDA) levels were quantified using 1-methyl-2-phenylindole (MPI) in an HCl-based assay. Briefly, 50 µL of the sonicated sample was mixed with MPI and incubated at 45 °C for 40 min. Absorbance was measured at 586 nm, and concentrations were calculated using a 1,1,3,3-tetramethoxypropane standard curve. For PCO content, samples were derivatized with 10 mM 2,4-dinitrophenylhydrazine. After adding 1 M NaOH, absorbance was measured at 450 nm. Both markers were normalized to total protein content.

### 2.9. Nuclear Morphology Assay and Morphological Cell Analysis

For fluorescence-based evaluation of condensed astrocyte nuclei, 1 × 10^5^ astrocyte cells were seeded in a 24-well plate. After reaching 90% confluence, cells were treated with glutamate alone or glutamate plus phenols. After 24 h, cells were fixed with 4% paraformaldehyde for 10 min at RT and then washed 3 times with PBS. Nuclei were stained with Fluorshield containing DAPI. Images were acquired using an epifluorescence microscope (OLYMPUS^®^ modular BX43, Tokyo, Japan). To determine the percentage of fragmented nuclei, 300 DAPI-stained nuclei were randomly selected per treatment group from multiple non-overlapping fields of view across three independent biological replicates. Nuclei were classified as healthy or apoptotic/fragmented based on chromatin condensation profiles and nuclear vesicle formation. All images were processed using ImageJ 1.44p software (NIH, Bethesda, MD, USA). For morphological cell analysis, bright-field images were captured using a NIKON^®^ ECLIPSE Ts2 inverted optical microscope. Random fields from different photographs were selected for each condition. Cell area and aspect ratio were determined using the formula: Aspect ratio = (Major axis)/(Minor axis). The region of interest (ROI) was selected from well-defined cells with complete morphology visible and no overlap, using the manual tool in ImageJ 1.44p (NIH, Bethesda, MD, USA).

### 2.10. qRT-PCR

Total RNA was isolated from C8-D1A astrocytes using TRIzol reagent following the manufacturer’s protocol. RNA concentration and purity were measured with a NanoDrop 2000 spectrophotometer (Thermo Fisher Scientific, Wilmington, DE, USA). cDNA was synthesized from 50 ng of RNA using the iScript™ Advanced cDNA Synthesis Kit for RT-qPCR. The 20 µL reverse transcription reaction was incubated in a Bio-Rad CFX96 thermal cycler at 46 °C for 20 min and then inactivated at 95 °C for 1 min. The resulting cDNA was quantified, and the expression of *IL-6*, *TNF-α*, *BDNF*, and *NGF* was measured by qPCR using the SsoAdvanced™ Universal SYBR Green Supermix (Bio-Rad, Hercules, CA, USA). Each 12.5 µL qPCR reaction included 6.25 µL of 2X Master Mix, 50 nM of each primer (see Supplementary Table S2), 60 ng of cDNA, and Milli-Q water. Amplification was performed on a Bio-Rad CFX96 Real-Time PCR System with an initial denaturation at 95 °C for 10 min, followed by 40 cycles of 95 °C for 15 s and 60 °C for 1 min. Gene expression was normalized to 18S RNA, and relative levels were calculated using the 2^−ΔΔCt^ method.

### 2.11. Western Blot Analysis

After treatment, astrocytes were lysed as previously described. Equal volumes of total protein were resolved by 10% SDS-PAGE and transferred to PVDF membranes. Membranes were blocked for 1 h at room temperature with TBST containing 5% Blotting-Grade Blocker. Membranes were then incubated overnight at 4 °C with constant agitation in TBST/1% Blocker containing the following primary antibodies: p-Akt (1:5000), total Akt (1:5000), p-Erk1/2 (1:5000), and anti-β-actin (1:5000), as previously described [26].

After washing, membranes were incubated with HRP-coupled secondary anti-mouse (1:10,000) or anti-rabbit (1:10,000) in TBST/1% Blocker for 1.5 h at room temperature. Proteins were detected using the Pierce^®^ ECL (Thermo Fisher Scientific, Waltham, MA, USA) Western Blotting Substrate on a ChemiDoc XRS+ (Bio-Rad). Images were processed and analyzed using ImageJ 1.44p software (NIH, Bethesda, MD, USA).

### 2.12. Statistical Analysis

All quantitative data are expressed as mean ± standard deviation (SD) from three independent experiments. Statistical comparisons were performed using one-way analysis of variance (ANOVA) followed by Dunnett’s post hoc test. Differences were considered statistically significant at *p* < 0.05. Statistical analyses were conducted using GraphPad Prism version 9.4 (GraphPad Software, San Diego, CA, USA).

## 3. Results

### 3.1. Glutamate-Induced Excitotoxicity Model in C8-D1A Astrocytes

**Figure 2:**
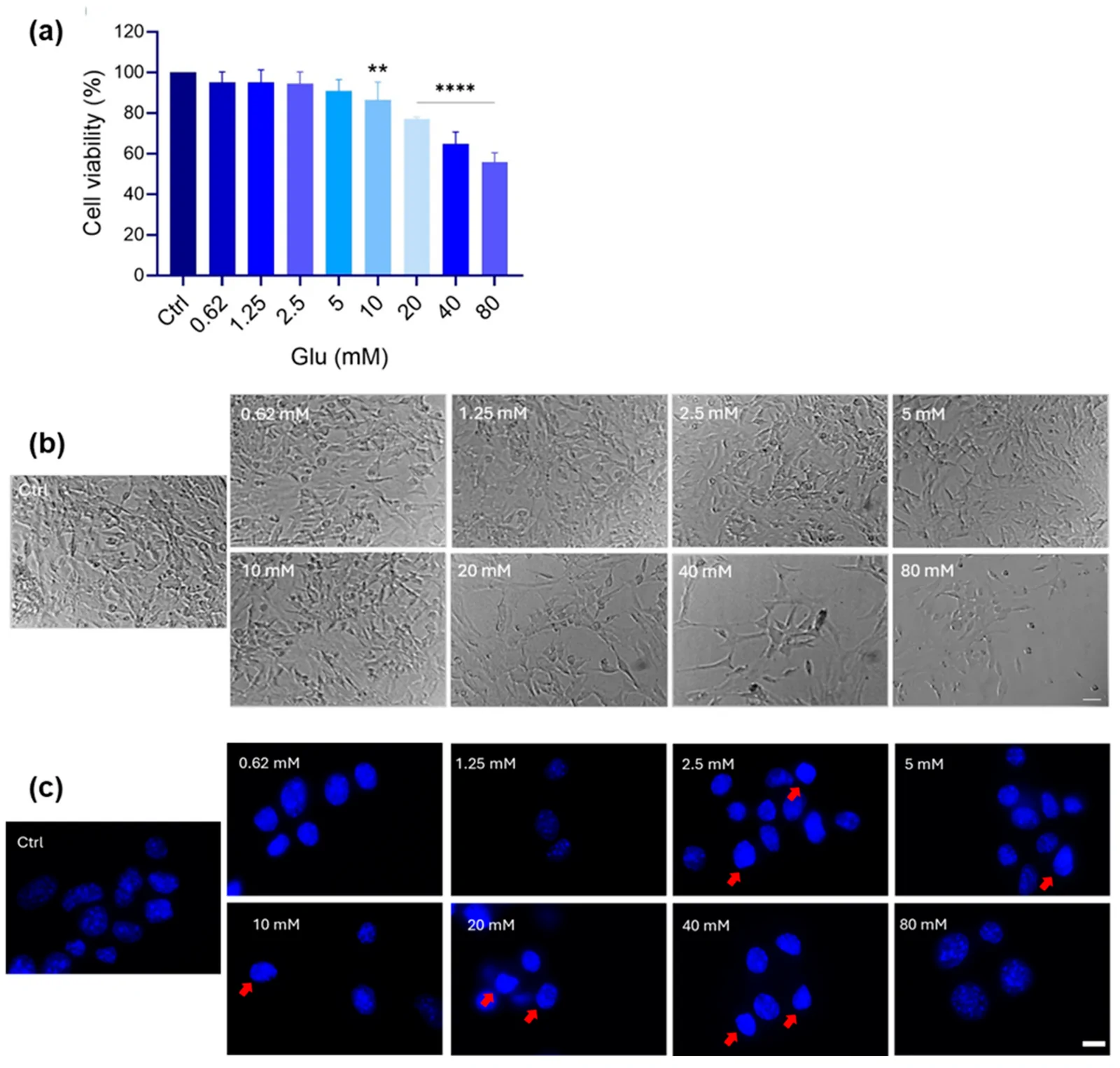
Evaluation of glutamate-induced excitotoxicity in C8-D1A astrocytes. Astrocytes were treated for 24 h with increasing concentrations of glutamate (Glu) (0.62 to 80 mM), and cell viability and imaging were assessed as previously described. (**a**) Cell viability was assessed by MTT assay; results are plotted as the mean ± SE of three independent experiments. Comparisons were made vs. untreated control cells (Ctrl). A one-way ANOVA was conducted, followed by Dunnett’s post hoc test; statistical significance was determined (** *p* < 0.001, **** *p* < 0.00001). (**b**) Representative brightfield images illustrating morphological changes, such as cell rounding and loss of prolongations, in Glu-treated cells. Images were obtained on a NIKON ^®^ ECLIPSE Ts2 microscope (Bar = 30 µm). (**c**) Representative fluorescence images (DAPI staining) obtained with an Olympus BX43 (Bar = 8 µm). Images show compromised nuclear integrity in Glu-treated cells, as evidenced by chromatin condensation (red arrows), compared with untreated controls. The original microscope images can be found in the Supplementary Materials.

To establish the Glu-induced damage model [25], C8-D1A astrocytes were exposed to 0.62–80 mM Glu for 24 h, and cell viability, morphology, and nuclear integrity were subsequently evaluated (Figure 2). Cell viability decreased starting at 10 mM Glu. Notably, at 20 mM, cell viability was reduced by approximately 20% (Figure 2a). Glu exposure induced a more circular cell shape, reduced cellular prolongations, and fewer cells per field compared with untreated control cells (Figure 2b). These alterations became progressively more pronounced as concentrations increased, indicating a concentration-dependent effect. Furthermore, Glu-treated cells exhibited compromised nuclear integrity, as evidenced by the detection of nuclear condensates (Figure 2c), a feature of ongoing apoptosis. Therefore, the 20 mM Glu concentration was selected as the optimal condition to induce moderate damage in the C8-D1A astrocyte cell line, as it effectively balances measurable cellular impairment with the preservation of sufficient viable cells for further evaluations.

### 3.2. Resorcinol, Hydroxyquinol, Hydroquinone, and Pyrogallol Prevent the Glu-Induced Damage in Astrocytes When Administered Simultaneously

**Figure 3:**
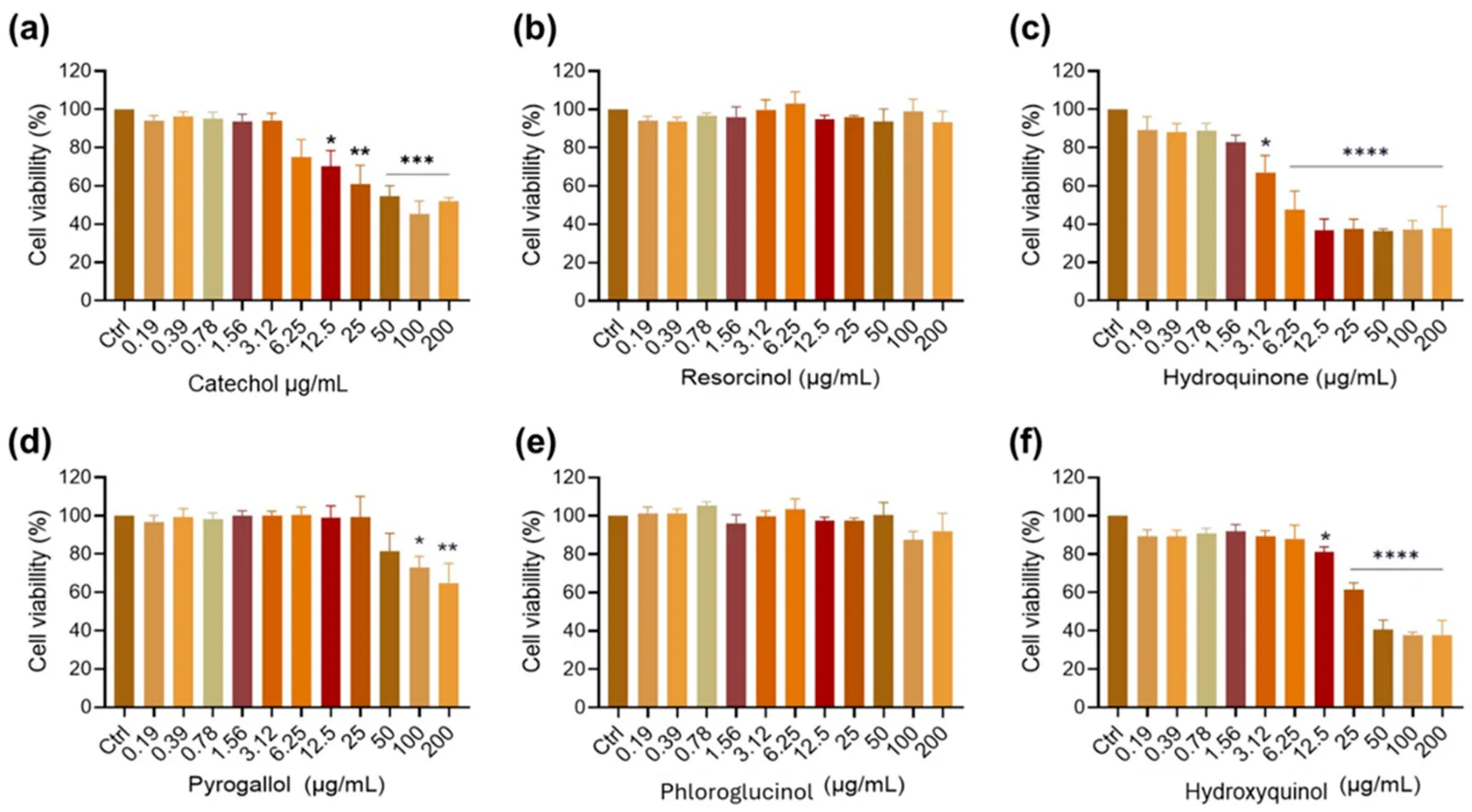
Effect of simple phenolic compounds on C8-D1A astrocyte cell viability. Cells were exposed for 24 h to increasing concentrations (0.19–200 µg/mL) of each phenolic compound, and an MTT assay was performed. Cell viability of (**a**) catechol, (**b**) resorcinol, (**c**) hydroquinone, (**d**) pyrogallol, (**e**) phloroglucinol, and (**f**) hydroxyquinol. Data are presented as mean ± SE of three independent replicates. Comparisons were made vs. the cell viability of untreated control cells (Ctrl). One-way ANOVA with Dunnett’s post hoc test; significance indicated as * *p* < 0.05, ** *p* < 0.001, *** *p* < 0.0001, **** *p* < 0.00001.

Simple phenols with two or three hydroxy groups exhibiting ortho-, meta-, and para-substitution patterns (Figure 1) were selected to test potential glioprotective bioactivity. Phenolic compounds can act as both antioxidants and pro-oxidants [18,19], and we describe how each affects the viability of C8-D1A astrocytes. We tested various concentrations of catechol, resorcinol, hydroquinone, pyrogallol, phloroglucinol, and hydroxyquinol, ranging from 0.19 to 200 µg/mL. The broad concentration range (0.19–200 µg/mL) was selected from a pilot test to identify the precise point at which these compounds transition from safe antioxidants to cytotoxic prooxidants (Figure 3). Our results show that catechol (≥6.25 µg/mL), hydroquinone (≥3.12 µg/mL), pyrogallol (≥50 µg/mL), and hydroxyquinol (≥25 µg/mL) significantly reduced cell viability (Figure 3a,c,d,f). Hydroquinone was the most cytotoxic compound, whereas resorcinol and phloroglucinol did not affect cell viability at any tested concentration (Figure 3b,e).

To evaluate the glioprotective potential of the compounds in the previously tested Glu-induced damage model, C8-D1A astrocytes were exposed to 20 mM Glu, and phenolic compounds were administered as pre-, co-, or post-treatments to determine which approach yielded the best results. Only resorcinol (≥3.12 µg/mL) and pyrogallol (0.78–25 µg/mL) administered as pre-treatments slightly improved cell viability compared with cells treated with Glu alone (Supplementary Figure S1b,d). However, none of the other compounds exhibited glioprotective effects when administered as pre-treatments (Supplementary Figures S1a,c,e,f). Subsequently, the six phenolic compounds were administered as post-treatments for an additional 24 h to Glu-damaged astrocytes to test whether they could mitigate the damage. Our results show that post-treatment failed to alleviate the decrease in cell viability caused by Glu exposure (Supplementary Figure S2). Moreover, catechol (≥50 µg/mL), hydroquinone (≥3.12 µg/mL), pyrogallol (≥25 µg/mL), and hydroxyquinol (≥3.12 µg/mL) exacerbated Glu-induced cellular damage (Supplementary Figures S2a,c,d,f).

**Figure 4:**
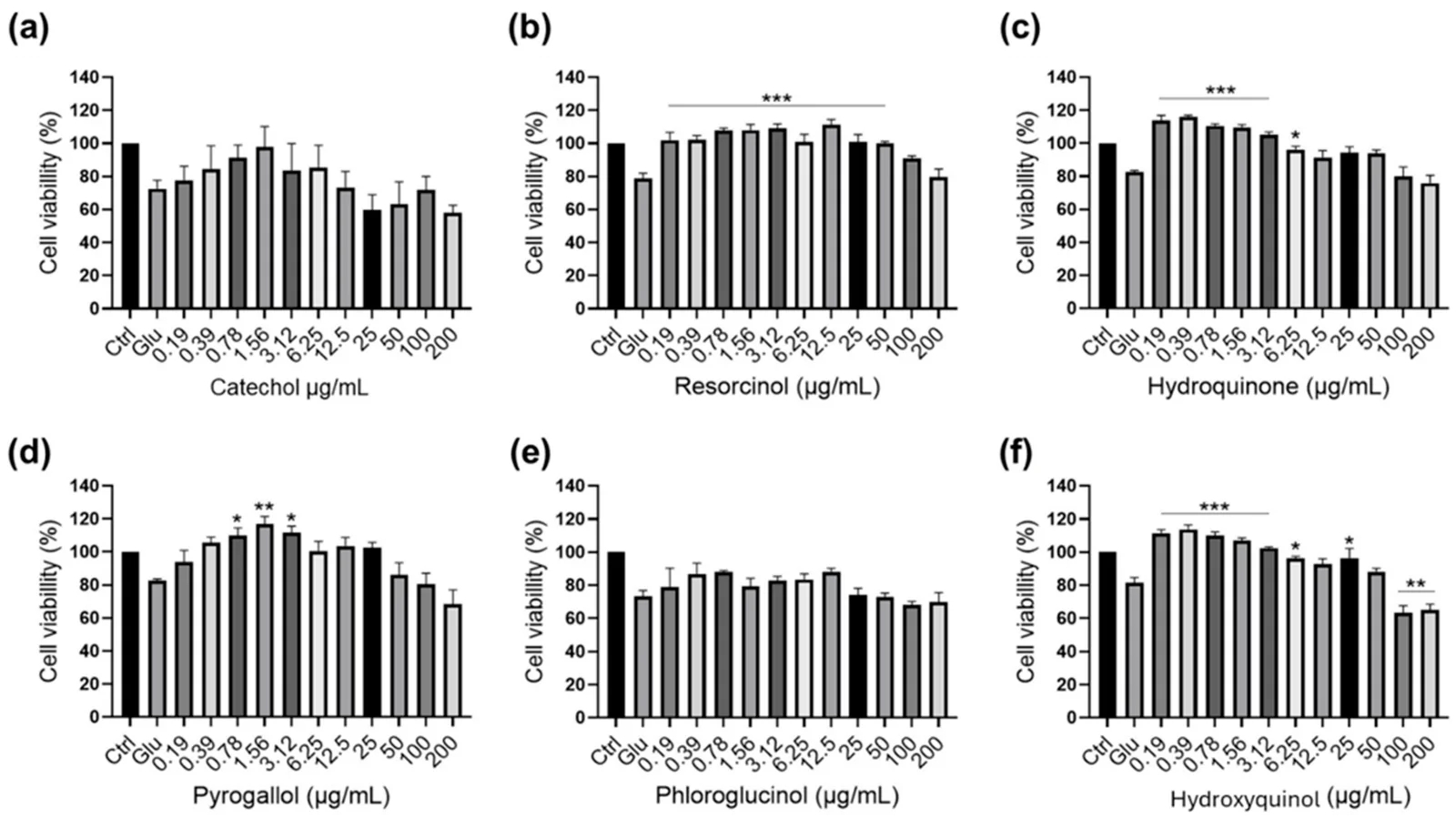
Glioprotective effects of simple phenols on Glu-treated C8-D1A astrocytes. Cells were simultaneously exposed to 20 mM Glu and varying concentrations (0.19–200 µg/mL) of each phenolic compound for 24 h, followed by an MTT assay. Cell viability was measured for (**a**) catechol, (**b**) resorcinol, (**c**) hydroquinone, (**d**) pyrogallol, (**e**) phloroglucinol, and (**f**) hydroxyquinol. Data are expressed as mean ± SE from three independent experiments. Comparisons were made against cells treated with Glu alone (Glu). One-way ANOVA with Dunnett’s post hoc test was performed; significance levels are indicated as follows: * *p* < 0.05, ** *p* < 0.001, *** *p* < 0.0001.

Next, 20 mM Glu and the phenolic compounds were administered simultaneously to C8-D1A astrocytes (Figure 4). This approach revealed a glioprotective effect, particularly with resorcinol, which, at concentrations of 0.19–100 µg/mL, consistently enhanced cell viability compared with Glu-alone treatments. The maximum protective effect of resorcinol was observed at 12.5 µg/mL (Figure 4b). Hydroquinone and hydroxyquinol enhanced cell viability at concentrations of 0.19–6.25 µg/mL (Figure 4c,f), whereas pyrogallol was effective at concentrations of 0.78–3.12 µg/mL (Figure 4d). Catechol and phloroglucinol did not alleviate Glu-induced damage (Figure 4a,e). Thus, among the six initial phenols, we selected resorcinol, hydroquinone, pyrogallol, and hydroxyquinol for further evaluation.

**Figure 5:**
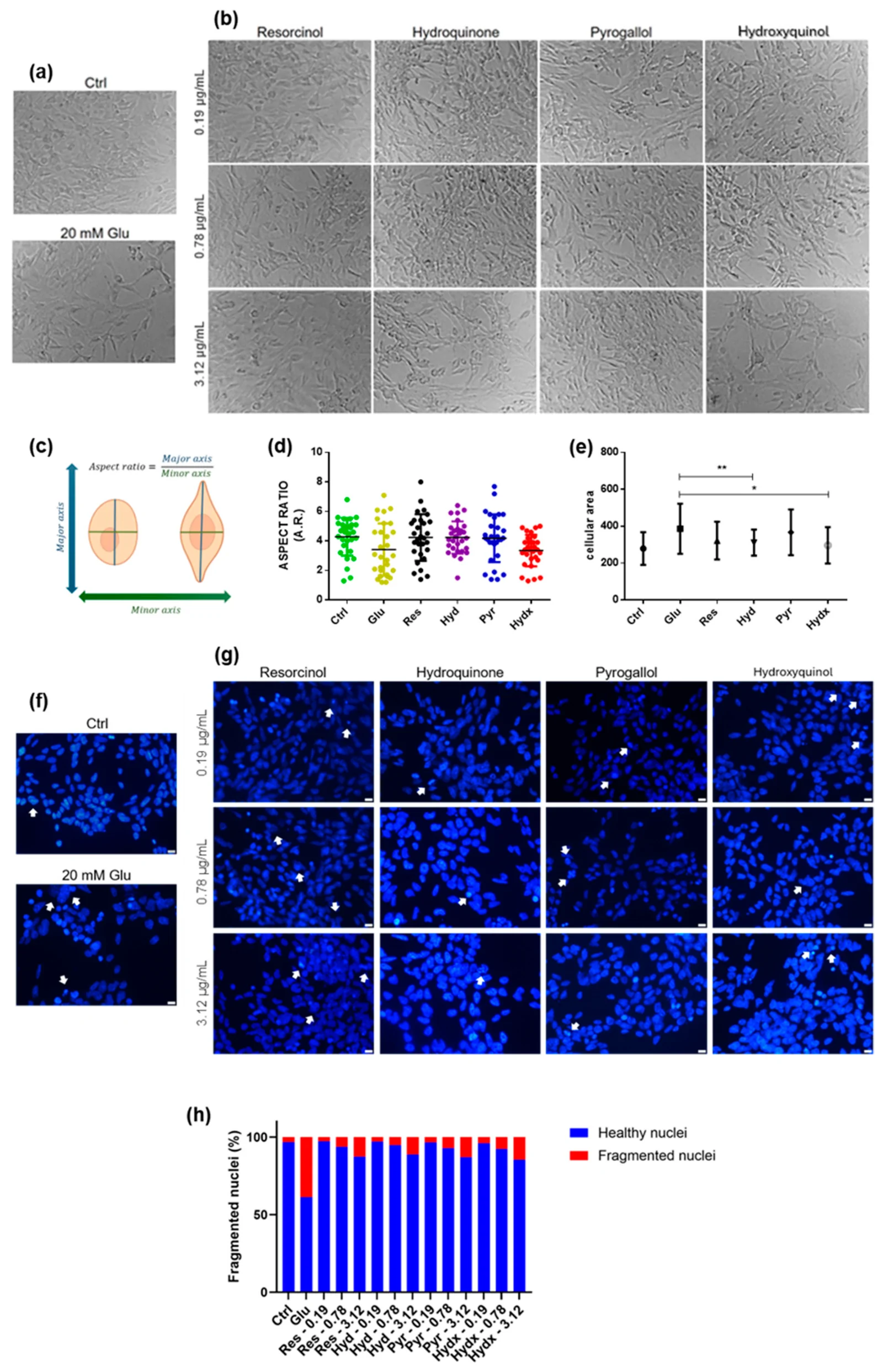
Morphological and nuclear protection in Glu-treated C8-D1A astrocytes in response to phenolic compounds. Cells were co-treated for 24 h with 20 mM Glu and the specified phenolic compounds at concentrations of 0.19, 0.78, and 3.12 µg/mL, after which brightfield and epifluorescence microscopy were performed. (**a**,**b**) Representative brightfield images show that astrocyte morphology remains unchanged in cells co-treated with phenols and Glu. Images were obtained on a NIKON^®^ ECLIPSE Ts2 microscope (Bar = 30 µm). (**c**) Schematic indicating the cell aspect ratio. (**d**) Analysis of the aspect ratio for individual cells. (**e**) Cell area for each experimental condition. Statistical significance was assessed using one-way ANOVA with Dunnett’s post hoc test (* *p* < 0.05, ** *p* < 0.001). (**f**,**g**) Representative epifluorescence images (DAPI staining) show reduced chromatin condensation in cells co-treated with phenols and Glu. White arrows indicate nuclear condensation. Images were obtained using an epifluorescence OLYMPUS^®^ BX43 microscope (Bar = 8 µm). (**h**) Quantitative analysis of nuclear morphology showing the percentage of healthy (blue bars) and fragmented/apoptotic (red bars) cell nuclei. Data represent the mean values from 3 independent experiments. The original microscope images are available in the Supplementary Materials.

Furthermore, the latter four phenolic compounds showed improved cellular morphology and nuclear integrity compared with cells exposed to Glu alone (Figure 5). In response to 0.19–3.12 µg/mL of resorcinol, hydroquinone, pyrogallol, and hydroxyquinol, a greater number of Glu-treated astrocytes maintained their distinctive cytoplasmic extensions without significant changes in size or shape (Figure 5a–d). Furthermore, Glu increases cell size; however, hydroquinone and hydroxyquinol prevent this morphological change (Figure 5e).

In addition, fewer cells exhibited nuclear condensation, as quantified by scoring fragmented nuclei in the presence of Glu and the four evaluated phenolic compounds (Figure 5f–h). These results suggest that, in our model, certain phenolic compounds, particularly resorcinol, can mitigate Glu-induced cytotoxicity and prevent morphological features of apoptosis, depending on concentration and administration strategy.

### 3.3. Oxidative Stress Relief in Glu/Phenols-Treated C8-D1A Astrocytes

**Figure 6:**
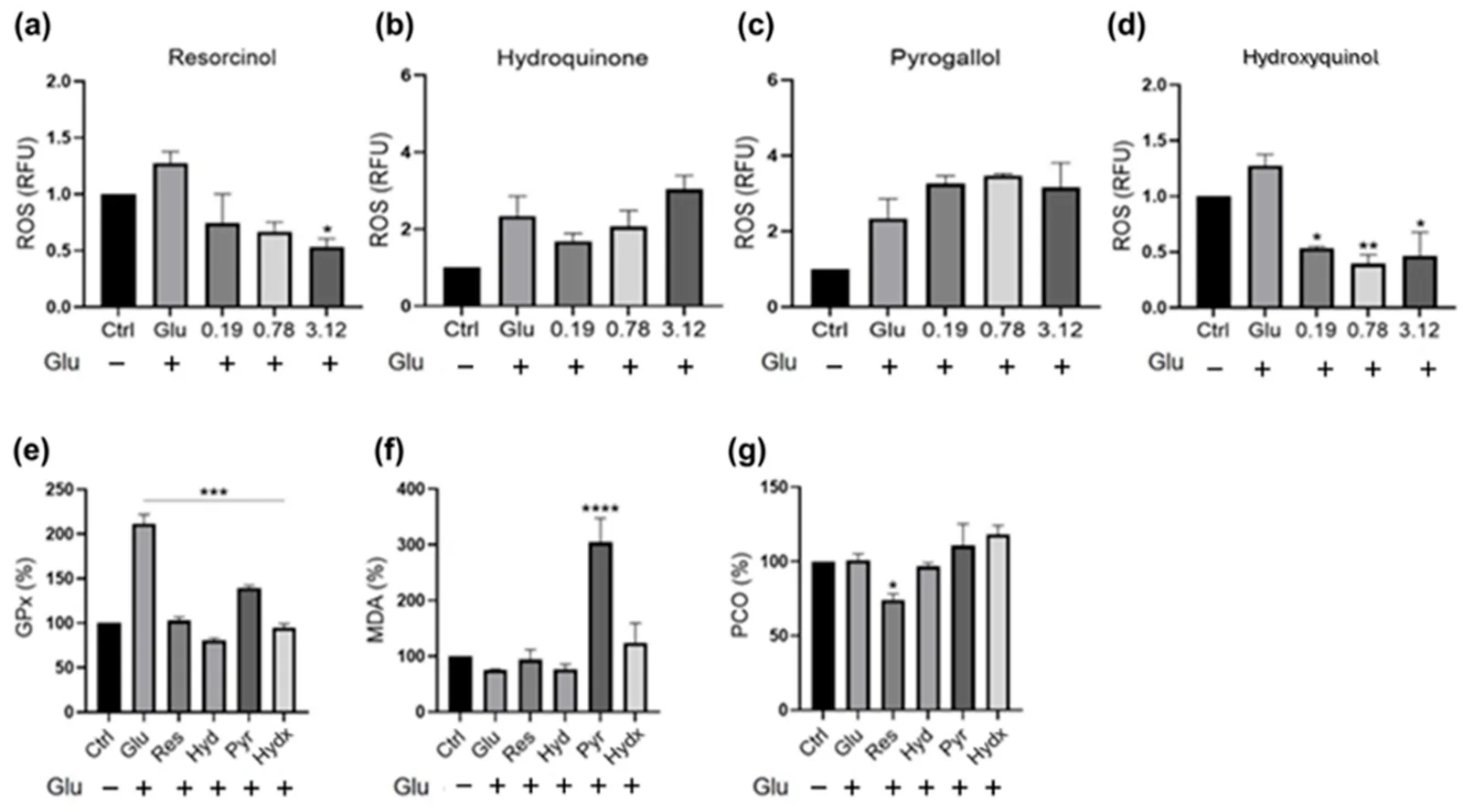
Oxidative stress markers in C8-D1A astrocytes treated with Glu/phenols. Cells were exposed simultaneously to 20 mM Glu and specific phenolic compounds, and oxidative stress-induced damage was measured. (**a**–**d**) Intracellular ROS levels were assessed using CellROX (resorcinol and hydroxyquinol) and DCFH-DA (hydroquinone and pyrogallol) after 3 h of treatment with 20 mM Glu plus 0.19, 0.78, and 3.12 µg/mL phenols. (**e**) Glutathione peroxidase (GPx) activity, (**f**) malondialdehyde (MDA) levels, and (**g**) protein carbonyl (PCO) content were determined in astrocytes treated with 20 mM Glu and 0.19 µg/mL phenols for 24 h. Data are expressed as mean ± SE from three independent experiments. Comparisons were made against cells treated with Glu alone (Glu). One-way ANOVA with Dunnett’s post hoc test; significance indicated as * *p* < 0.05, ** *p* < 0.001, *** *p* < 0.0001, and **** *p* < 0.00001.

Resorcinol, hydroquinone, pyrogallol, and hydroxyquinol alleviate Glu-induced reductions in cell viability and morphological damage in C8-D1A astrocytes. To determine whether this effect was linked to the antioxidant properties of phenols, we measured antiradical capacity, reactive oxygen species (ROS) levels, lipid peroxidation (MDA), protein carbonylation (PCO), and glutathione peroxidase (GPx) activity (Figure 6). Although resorcinol (Res) showed the greatest protective effect at 12.5 µg/mL, viability tests revealed that hydroquinone (Hyd), pyrogallol (Pyr), and hydroxyquinol (Hydx) became highly cytotoxic at 6.25 µg/mL or higher. Consequently, the 0.19–3.12 µg/mL range represents the largest common protective window, enabling a direct comparison of all four compounds without the confounding effects of significant cell death. Accordingly, oxidative stress markers were measured within this range to ensure consistent testing conditions across all four phenolic compounds.

Despite their positive impact on cell viability, hydroquinone and pyrogallol did not reduce ROS levels (Figure 6b,c); moreover, pyrogallol strongly increased MDA levels, indicating greater lipid membrane damage (Figure 6f).

On the other hand, resorcinol or hydroxyquinol/Glu-cotreated cells showed significantly lower ROS levels than those treated with Glu alone (Figure 6a,d); both restored the Glu-induced GPx activity peak to baseline levels (Figure 6e), and interestingly, only resorcinol showed a reduction in the Glu-induced protein oxidation (PCO) (Figure 6g). This suggests that the observed glioprotective effects of these compounds are likely mediated by their radical-scavenging and antioxidant activities (Supplementary Table S1).

### 3.4. Immunomodulation and Neurotrophic Factor Expression in Glu/Phenols-Treated C8-D1A Astrocytes

**Figure 7:**
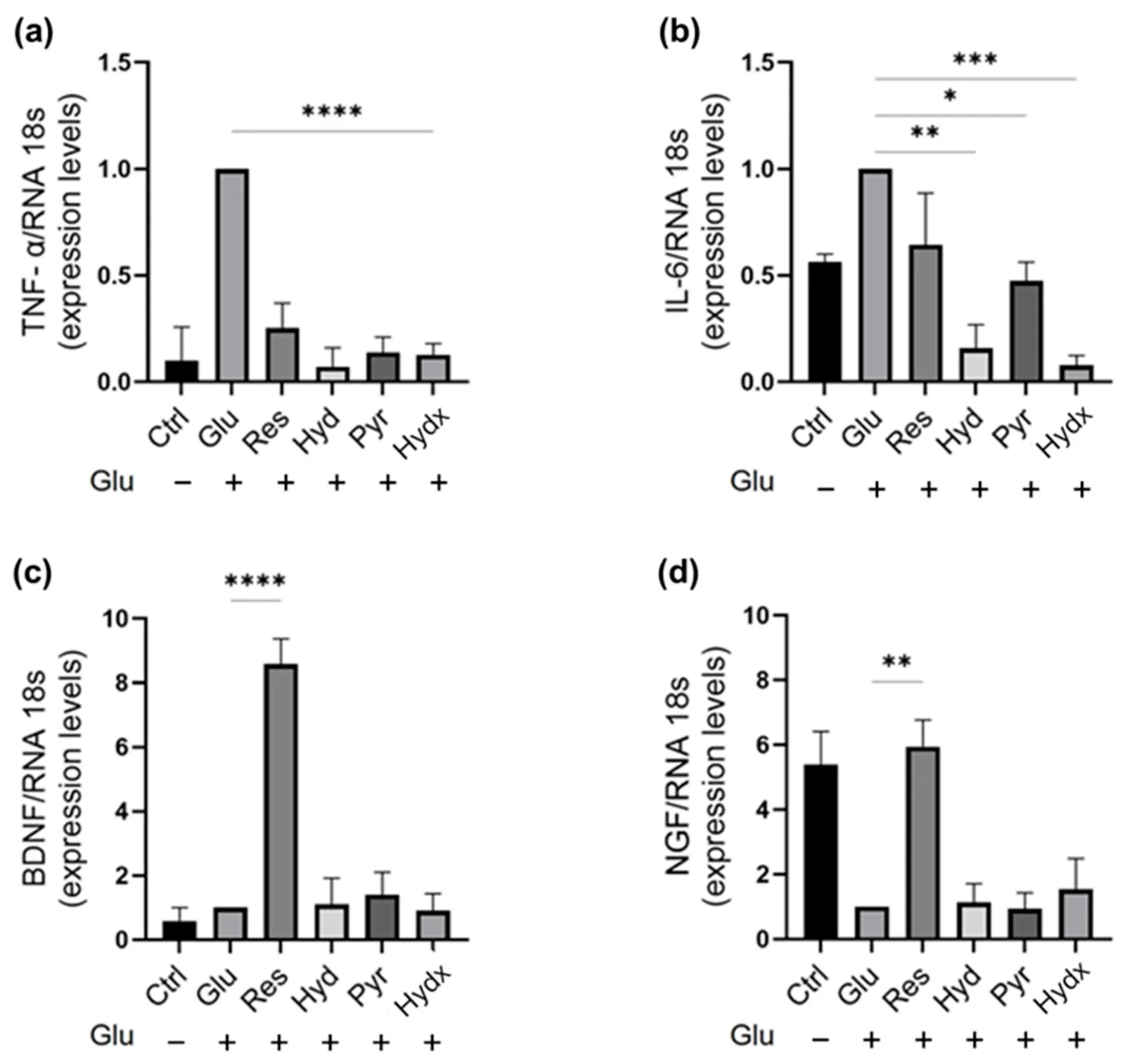
Effects of phenolic compounds on proinflammatory cytokine and neurotrophic factor expression in glutamate-treated astrocytes. Relative gene expression was measured by RT-qPCR in C8-D1A astrocytes treated simultaneously with 20 mM Glu and phenolic compounds (0.19 µg/mL) for 24 h. Expression levels of proinflammatory cytokines (**a**) *TNF-α* and (**b**) *IL-6*. Expression of neurotrophic factors (**c**) *BDNF* and (**d**) *NGF.* Data are presented as mean ± SE from three independent experiments. Gene expression was normalized to 18S RNA and quantified using the 2^−ΔΔCt^ method. Comparisons were made vs. Glu-only-treated cells (Glu). A one-way ANOVA was conducted, followed by Dunnett’s post hoc test; statistical significance was denoted as * *p* < 0.05, ** *p* < 0.001, *** *p* < 0.0001, and **** *p* < 0.00001.

The neuroprotective capacity of phenolic compounds is largely attributed to their anti-inflammatory properties and their ability to modulate cell signaling [27]. Accordingly, we evaluated the expression of the proinflammatory cytokines *TNF-α* and *IL-6*, which were overexpressed in Glu-damaged astrocytes. All four selected simple phenols robustly downregulated *TNF-α* relative to Glu-treated cells (Figure 7a). Hydroquinone and hydroxyquinol induced 90% and 85% downregulation of *IL-6*, respectively. Similarly, resorcinol (37%), pyrogallol (52%), hydroquinone (85%), and hydroxyquinol (92%) significantly reduced *IL-6* expression (Figure 7b). Decreased cytokine levels, modulation of survival pathways, and increased expression of neurotrophic factors have been implicated in neuroprotective processes [28,29]. Therefore, we evaluated the expression of two neuroprotective neurotrophins, nerve growth factor (NGF) and brain-derived neurotrophic factor (BDNF). Glu-treated cells showed low *NGF* levels. Regarding neurotrophic factors, resorcinol markedly increased *BDNF* expression compared with Glu-treated cells, whereas the other compounds produced only minor changes (Figure 7c). In contrast, the Glu-induced reduction in *NGF* expression was partially reversed by resorcinol, resulting in a significant increase relative to Glu-treated cells, whereas pyrogallol, hydroquinone, and hydroxyquinol did not significantly affect *NGF* levels (Figure 7d).

**Figure 8:**
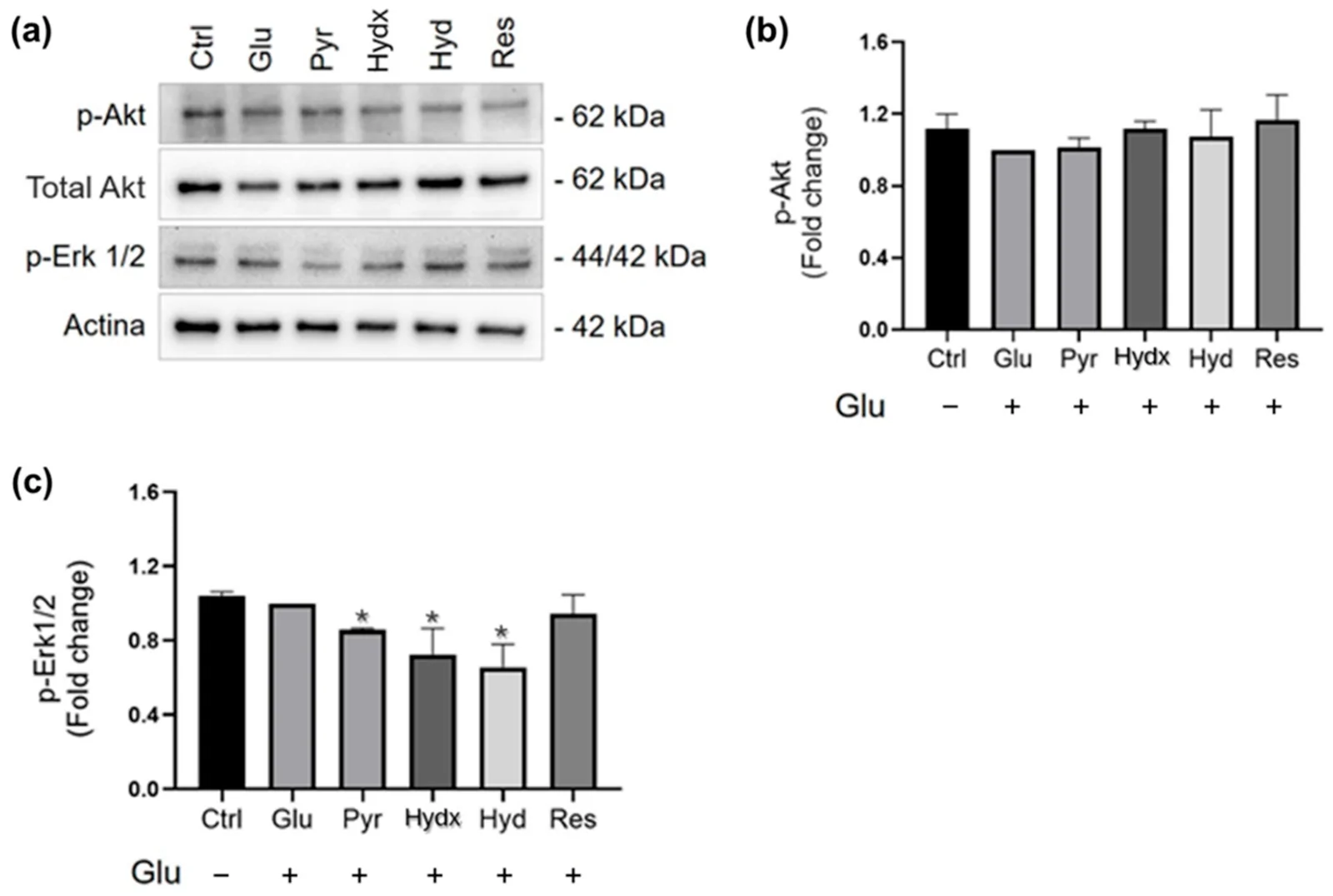
Modulation of ERK1/2 and Akt phosphorylation by phenolic compounds in glutamate-treated astrocytes. Phosphorylation of Akt and ERK1/2 was assessed by Western blot in C8-D1A astrocytes treated simultaneously with 20 mM Glu and phenolic compounds (0.19 µg/mL) for 24 h. (**a**) Western blot of p-Akt, total Akt, p-ERK1/2, and β-actin. Densitometric analysis showed fold change in phosphorylation of (**b**) Akt and (**c**) ERK1/2, with activation normalized to β-actin expression. The P-Akt signal was normalized to total Akt. Data are expressed as mean ± SE of three independent experiments. Comparisons were made vs. Glu-only-treated cells (Glu). A one-way ANOVA with Dunnett’s post hoc test was conducted, with significance considered at * *p* < 0.05. The original images of the western blot can be found in the Supplementary Materials.

To determine whether the protective effects of the phenolic compounds were associated with activation of pro-survival signaling pathways, the phosphorylation levels of Akt and ERK1/2 were evaluated in C8-D1A astrocytes exposed to glutamate. As shown in Figure 8a, no evident changes in Akt phosphorylation were observed across the different treatments. Densitometric analysis confirmed that p-Akt levels remained comparable to those detected in glutamate-treated cells (Figure 8b). In contrast, glutamate-treated cells showed significantly reduced ERK1/2 phosphorylation compared with untreated controls. Among the phenolic compounds evaluated, resorcinol partially restored ERK1/2 phosphorylation levels, whereas pyrogallol, hydroxyquinol, and hydroquinone did not produce significant changes relative to glutamate-treated cells (Figure 8c). These results suggest that the mechanisms underlying the bioactivity of phenols may involve modulation of the ERK1/2 signaling pathway.

## 4. Discussion

Recent research has reinforced the view that astrocytes actively regulate glutamate balance rather than merely serving as support cells. Glutamate imbalance can lead to oxidative stress, neuroinflammation, and mitochondrial dysfunction, making astrocytes key targets for neuroprotection [5,30]. Additionally, modulating glutamatergic signaling and oxidative stress has become a primary strategy to reduce excitotoxic damage in neurodegenerative diseases.

Neuronal and astrocytic loss due to Glu-induced excitotoxicity is a hallmark of neurodegeneration [2]. Therefore, we aimed to induce measurable cellular impairment without completely compromising the survival capacity of C8-D1A astrocytes, thereby enabling further evaluation. We established a model in which Glu reduced cell viability, accompanied by mild nuclear condensation and morphological changes, features characteristic of apoptotic cells. We then implemented three protocols for administering six simple phenols (pre-, co-, and post-treatment), as previously described [31].

Our results show that meta-substituted phenols, such as resorcinol and phloroglucinol, are more biocompatible than ortho- and para-substituted phenols, including catechol, hydroquinone, pyrogallol, and hydroxyquinol, which significantly decreased cell viability at 6.25 µg/mL or higher. At low-to mid-concentrations, resorcinol and pyrogallol slightly enhanced cell viability when used as pre-treatments in Glu-damaged astrocytes. None of the six phenols reduced Glu-induced cytotoxicity when applied after damage. Additionally, the cytotoxic ortho- and para-phenols worsened Glu damage.

These results show that (a) pre-treatment with phenols does not prevent Glu-induced damage, (b) post-treatment cannot reverse it, and (c) phenols with ortho- and para-configurations act as pro-oxidants when administered alone or after Glu. Evidence indicates that phenols with -OH groups in ortho- and para-positions are more stable than meta-ones, and ortho-diphenolics show strong antioxidant capacity [32]. However, as observed, the hyperreactivity of these phenols can trigger pro-oxidant effects, possibly due to reactive semiquinone radicals. In the presence of Cu^2+^, Fe^3+^, and O_2_, some phenols can generate phenoxy radicals that induce lipid peroxidation and DNA damage [33] and inhibit glutathione transferase, thereby worsening cytotoxicity [34].

The pro-oxidant properties of phenols largely originate from hyperreactivity and/or potent scavenging capabilities, as exemplified by catechol and pyrogallol (Supplementary Table S1). This underscores their dualistic nature, in which antioxidant effects may shift to pro-oxidant effects under specific conditions. Our research demonstrates that resorcinol, hydroquinone, pyrogallol, and hydroxyquinol enhance cell viability and maintain cellular morphology along with nuclear integrity in C8-D1A cells, even in the presence of Glu. Further investigation is warranted to elucidate the mechanisms through which certain phenolics, such as catechol and phloroglucinol, contribute to cellular damage during glutamate-induced excitotoxicity.

Despite their positive effects on cell viability, hydroquinone and pyrogallol slightly increased ROS levels. Their ability to improve astrocyte survival, despite no significant reduction in intracellular ROS, suggests other protective mechanisms, such as modulating apoptotic signaling, maintaining redox balance, or activating cytoprotective pathways, though these were not studied here. Pyrogallol closely matched Glu-induced GPx activity, caused slight protein carbonylation, and increased MDA levels, indicating lipid damage. These pro-oxidant effects likely relate to its reactivity (IC50 < 3.12 µg/mL), as semiquinones react with protein residues [35], generating hydroxyl radicals that promote lipid peroxidation [36,37]. This behavior exemplifies a typical cellular hormetic response [38]. At very low doses (0.19 µg/mL), pyrogallol’s unstable structure rapidly undergoes autooxidation, resulting in minor, localized lipid damage, as evidenced by an MDA spike. This sublethal stress serves as a preconditioning signal, boosting the cell’s natural defenses and making astrocytes more resistant to higher levels of excitotoxicity. Similarly, although hydroquinone is highly cytotoxic due to its unstable para-hydroxyl groups, co-treatment with Glu changes its reaction kinetics. This allows it to temporarily act as a chemical buffer against excitotoxicity before its toxicity becomes evident. In contrast, resorcinol and hydroxyquinol significantly reduced ROS levels and restored GPx activity in astrocytes damaged by Glu. Resorcinol uniquely reduced protein oxidation.

The four phenols analyzed in this study (resorcinol, hydroquinone, pyrogallol, and hydroxyquinone) exhibited significant anti-inflammatory activity in glial cells, as previously described for other phenols [11,12,32]. The marked attenuation of proinflammatory cytokines (*IL-6* and *TNF-α*) may be mechanistically linked to modulation of the ERK1/2 pathway, as the MAPK/ERK cascade is a critical regulator of inflammatory mediator transcription in glial cells [39]. Furthermore, in astrocytes, unlike in neurons, severe glutamate-induced excitotoxicity triggers prolonged pathological overactivation of ERK1/2, which strictly promotes apoptosis rather than survival [40]. By preventing or modulating this glutamate-induced ERK overactivation, simple phenols short-circuit the inflammatory cascade before it can drive pathological gene expression. Our findings are consistent with recent paradigms highlighting that modulation of glial activation and its subsequent neuroinflammatory cascade constitutes a potential therapeutic target that, with further study, may prove crucial for slowing chronic neurodegeneration [41].

In the present C8-D1A astrocyte model, exposure to 20 mM glutamate decreased NGF expression, suggesting that severe glutamate-induced stress may compromise astrocytic neurotrophic support. This response appears to depend on the intensity and duration of the insult, as lower or sublethal glutamate exposure has been reported to stimulate NGF expression in other glial models [42]. Hydroquinone, pyrogallol, and hydroxyquinol had minimal effects on *BDNF* and *NGF* expression, indicating that their protective mechanisms primarily mitigate neuroinflammation rather than enhance neurotrophic gene expression.

Resorcinol not only has antioxidant properties but also diminishes Glu-induced protein oxidation, downregulates the proinflammatory cytokine *TNF-α*, and significantly upregulates *BDNF* and *NGF*. These effects indicate that its benefits also originate from anti-inflammatory properties and the promotion of neurotrophic mechanisms.

Evidence indicates that neurotrophins such as BDNF and NGF, along with the Trk/Akt pathway, are involved in cell survival [27,28,29]. To assess glioprotection in the glutamate injury model, we evaluated activation of Akt and ERK1/2.

Our results showed that Glu-damaged cells did not exhibit significant increases in Akt (p-Akt) or ERK1/2 (p-ERK1/2) activation, remaining similar to untreated controls. Most phenolic treatments did not markedly promote Akt activation, although resorcinol slightly increased p-Akt levels. Akt activation, linked to cell survival, especially in the central nervous system, is associated with the expression of glutamate transporters such as GLT-1, which help remove excess Glu [40]. We hypothesized that resorcinol might have a similar effect in our model. ERK1/2 kinase has a dual role: generally promoting survival, but prolonged ERK activation in astrocytes can trigger apoptosis [43]. Our data did not show significant ERK activation in the Glu-damaged model, likely because the Glu concentration used does not sustain ERK activation, unlike concentrations ≥ 50 mM Glu [44,45]. However, we observed that pyrogallol, hydroxyquinol, and hydroquinone slightly decreased p-ERK1/2 levels relative to Glu-treated cells, suggesting that these compounds may confer neuroprotection by modulating ERK1/2 activity, unlike resorcinol. Although resorcinol partially restored ERK1/2 phosphorylation, our findings do not prove that ERK activation is crucial for glioprotection. Further studies using inhibitors are needed to clarify whether ERK signaling is causal. The lack of significant Akt phosphorylation suggests that the PI3K/Akt pathway may not be the primary protective mechanism. Other pathways, such as Nrf2/ARE, NF-κB, and CREB, which regulate antioxidant responses and inflammation, might also be involved [46,47,48,49].

Among the compounds tested, resorcinol showed the most consistent glioprotective activity, while hydroxyquinol, hydroquinone, and pyrogallol also enhanced astrocyte viability when co-administered with glutamate at low doses. These effects were correlated with reduced oxidative stress and inflammation. The findings underscore that simple phenolic compounds may act as either antioxidants or pro-oxidants, depending on their molecular structure and exposure conditions. Nonetheless, the study has certain limitations: all experiments used a single immortalized mouse astrocyte cell line (C8-D1A), which may not fully represent primary astrocyte responses or the complexity of the CNS; the research was confined to in vitro assessments, necessitating further validation; furthermore, despite evaluating antioxidant and anti-inflammatory responses, the intracellular pathways involved were not examined.

Future research should investigate the molecular mechanisms underlying the protective effects of simple phenols, including the PI3K/Akt, MAPK/ERK, and Nrf2 signaling pathways, as well as apoptosis-related proteins such as Bcl-2, Bax, and caspase-3. Validation in primary astrocytes, neuron–astrocyte co-cultures, and in vivo models will be essential to confirm their neuroprotective potential. In addition, pharmacokinetic studies, blood–brain barrier (BBB) permeability, and toxicological evaluation are required before clinical translation, since BBB penetration depends not only on molecular weight but also on lipophilicity, metabolism, transporter interactions, and plasma protein binding [50]. Resorcinol appears to be a promising scaffold for SAR because of its favorable neuroprotective profile and relatively low redox reactivity, although its rapid metabolism may limit brain exposure [51]. In contrast, the redox activity of hydroquinone and the propensity of pyrogallol and hydroxyquinol to undergo autooxidation and generate ROS may restrict their therapeutic applicability unless these limitations are addressed through structural optimization [52].

Our model shows a meaningful SAR, as ortho-, meta-, and para-substitutions on phenols affect the outcomes. Hydroquinone and hydroxyquinol’s ortho- and para-forms promote survival and antioxidant activity but become prooxidants at higher concentrations, likely due to electrophilic substitution at accessible OH groups. Conversely, the meta-resorcinol, with slower electrophilic substitution, offers better buffering during redox reactions and forms stronger, more specific protein interactions [53,54].

Hydroxyl- and alkyl-substituted phenols, such as cresols, highlight SAR differences. Hydroxyl groups influence redox potential through resonance and induction, whereas methyl groups donate electrons, increasing electron density and potentially accelerating oxidation. Hydroxyls primarily affect redox via hydrogen transfer, whereas methyl groups alter lipophilicity and membrane affinity, thereby influencing antioxidant or pro-oxidant activity [55,56].

Although phloroglucinol has the same meta-substitution as resorcinol, it showed no significant bioactivity in C8-D1A astrocyte excitotoxicity tests. The meta-OH placement provides chemical stability and prevents autooxidation, but the tri-hydroxyl arrangement might cause steric hindrance or alter interactions with target proteins or Glu receptors [21,57]. This suggests that the degree of hydroxylation, not just the pattern, is key to glioprotective activity.

## 5. Conclusions

Resorcinol, a meta-substituted compound, effectively safeguards glial cells by promoting their survival, acting as an antioxidant and anti-inflammatory agent, and elevating neurotrophic factor levels. It exhibits no cytotoxicity even at elevated concentrations. This research may provide valuable insights for future investigations into how resorcinol mitigates Glu-induced oxidative damage and neuroinflammation in preclinical studies.

Our results demonstrate that the spatial configuration of phenolic hydroxyl groups influences bioactivity, with the meta-configuration (resorcinol) showing more pronounced neuroprotective effects. The study supports the hypothesis that simple phenolic compounds may mitigate glutamate-induced damage and underscores the importance of SAR in developing efficacious bioactive agents.

## Data Availability

The original contributions presented in this study are included in the article/Supplementary Material. Further inquiries can be directed to the corresponding authors.

## Supplementary Materials

The following supporting information can be downloaded at: https://www.mdpi.com/article/10.3390/jox16040142/s1. Figure S1. Effects of pre-treatment with simple phenolic compounds on the cell viability of Glu-treated C8-D1A astrocytes. Figure S2. Effects of post-treatment with simple phenolic compounds on the cell viability of Glu-treated C8-D1A astrocytes. Table S1. IC50 of simple phenols scavenging DPPH and ABTS synthetic radicals. Table S2. Primer sets for RT-qPCR.
