# Supplementary material for: Resorcinol and Related Simple Phenolic Compounds Increase Astrocyte Survival in a Glutamate-Induced Excitotoxicity Model
Source: J Xenobiot. 2026 Jul 31;16(4):142. doi: 10.3390/jox16040142 (PMC13514331; doi:10.3390/jox16040142)
Supplement: Supplementary file 1 [file jox-16-00142-s001.zip › jox-4415064-supplementary.pdf]

Supplementary Materials: Resorcinol and Related Simple Phenolic Compounds Increase Astrocyte Survival in a Glutamate-Induced Excitotoxicity Model

José M. Nájera-Maldonado [1], Paola Molina-Manzano [1], Eugenia Flores-Alfaro [1], Mónica Espinoza-Rojo [1], Isela Parra-Rojas [1], Patricia Alvarez-Fitz [2], Mónica Lamas [3], Ricardo Salazar [2,*] and Mónica Ramírez [2,*]

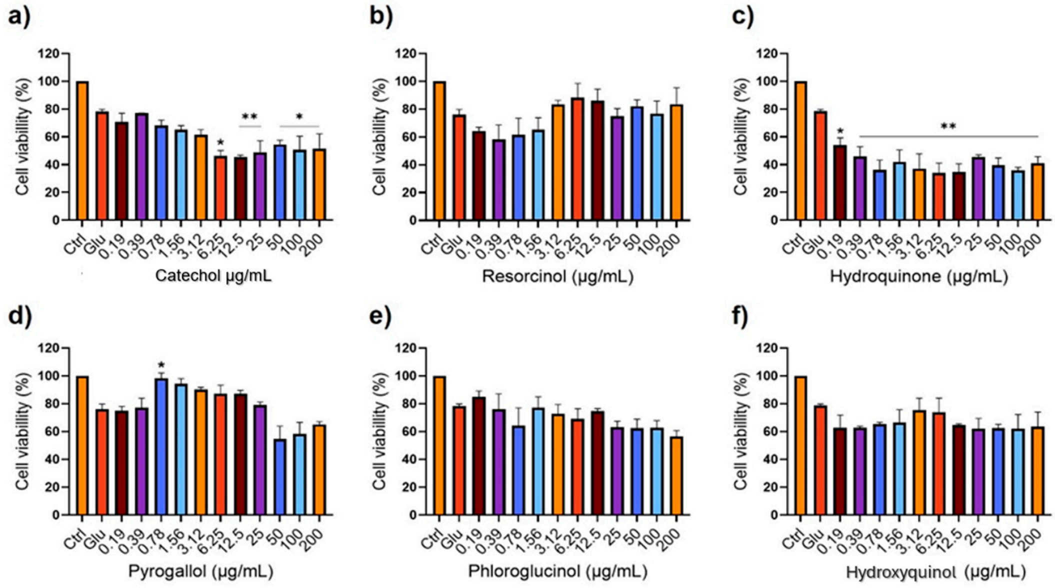

Figure S1. Effects of pre-treatment with simple phenolic compounds on the cell viability of Glu-treated C8-D1A astrocytes. Cells were pre-treated with increasing concentrations (0.19–200 µg/mL) of each phenolic compound for 24 h, then 20 Glu was added for an additional 24 h, and finally the MTT assay was performed. Cell viability of (a) catechol, (b) resorcinol, (c) hydroquinone, (d) pyrogallol, (e) phloroglucinol, and (f) hydroxyquinol. Data are presented as mean ± SE of three independent replicates. Comparisons were made vs. the cell viability of untreated control cells (Ctrl). Significance (*$p<0.05$, **$p<0.001$) was assessed by one-way ANOVA with Dunnett's post hoc test.

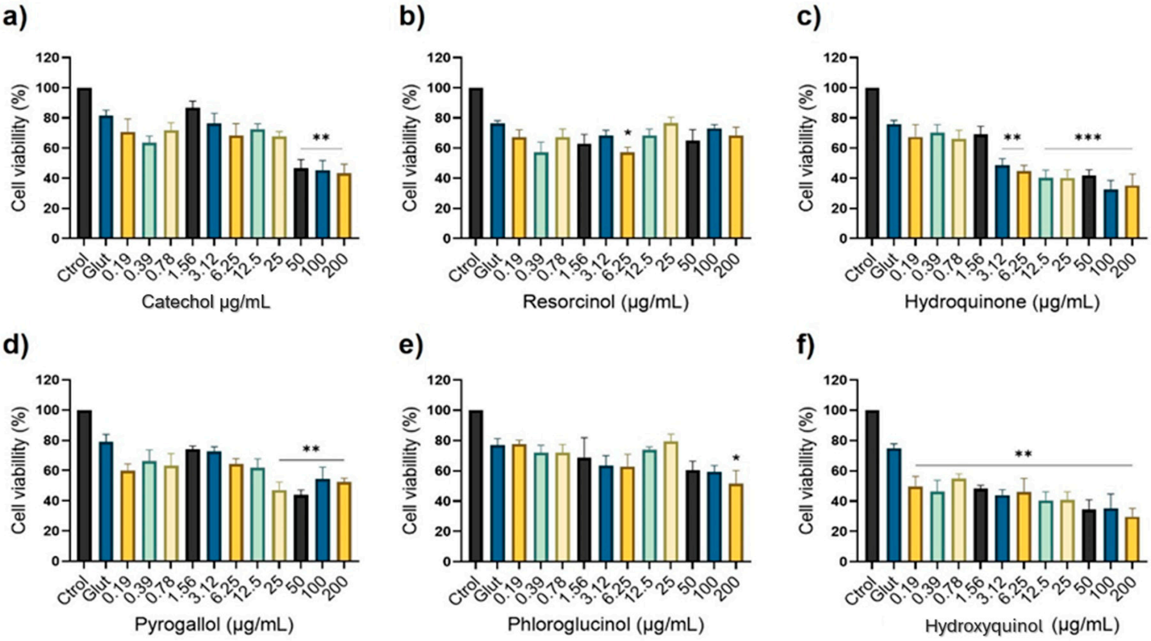

Figure S2. Effects of post-treatment with simple phenolic compounds on the cell viability of Glu-treated C8-D1A astrocytes. Cells were exposed to 20 mM Glu, then increasing concentrations (0.19–200 µg/mL) of each phenolic compound were added as post-treatments for an additional 24 h, and finally the MTT assay was performed. Cell viability of (a) catechol, (b) resorcinol, (c) hydroquinone, (d) pyrogallol, (e) phloroglucinol, and (f) hydroxyquinol. Data are presented as mean ± SE of three independent replicates. Comparisons were made vs. the cell viability of untreated control cells (Ctrl). Significance (*p<0.05, **p<0.001, ***p<0.0001) was assessed by one-way ANOVA with Dunnett's post hoc test.

Table S1. IC_{50} of simple phenols scavenging DPPH and ABTS synthetic radicals.

	IC_{50} (µg/mL)	
Compound	DPPH	ABTS
Catechol	>200	4.27
Resorcinol	203	7.56
Hydroquinone	206	<12.5
Pyrogallol	221	<3.12
Phloroglucinol	9.98	11.22
Hydroxyquinol	208	24.36

IC_{50} value (mean inhibitory concentration).

Table S2. Primer sets for RT-qPCR.

Gene	Primer sets
NGF	Forward: CAGTGTCAGTGTGTGGGTTG

	Reverse:<br>TGTGAGTCGTGGTGCAGTAT<br>PRODUCT: 206 pb
BDNF	Forward:<br>TGCAGGGGCATAGACAAAAGG<br>Reverse:<br>CTTATGAATCGCCAGCCAATTCTC<br>PRODUCT: 154 pb
TNF-α	Forward:<br>AGCCGATGGGTTGTACCTTG<br>Reverse:<br>ATAGCAAATCGGCTGACGGT<br>PRODUCT: 99 pb
IL-6	Forward:<br>ATCCAGTTGCCTTCTTGGGACTGA<br>Reverse:<br>TAAGCCTCCGACTTGTGAAGTGGT<br>PRODUCT: 134 pb
